# Editorial: The role of imaging in gynecological malignancies

**DOI:** 10.3389/fonc.2023.1238537

**Published:** 2023-06-22

**Authors:** Kathy Han, Alessio G. Morganti

**Affiliations:** ^1^ Department of Radiation Oncology, Princess Margaret Cancer Centre, University Health Network, University of Toronto, Toronto, ON, ;Canada; ^2^ Radiation Oncology, IRCCS Azienda Ospedaliero-Universitaria di Bologna, Bologna, Italy; ^3^ Radiation Oncology, Department of Medical and Surgical Sciences (DIMEC), Alma Mater Studiorum - Bologna University, Bologna, Italy

**Keywords:** editorial, gynecological neoplasms, imaging, cervical cancer, MRI

Despite the improvements recorded in the last decades, the outcome of patients with gynecological cancer is still unfavorable, in particular for some tumors and for some risk groups. At the same time, the evolution of imaging techniques in oncology is progressively accelerating, with the growing importance of topics such as the application of artificial intelligence and the study of body composition. The papers included in this Research Topic present an interesting summary of novel and still controversial aspects in the field of imaging of gynecological tumors (Scepanovic et al.; Hu et al.; Wang et al.; Matsuura et al.; Zhu et al.; Rizzo et al.; Cordoba et al.; Matani et al.; deSouza et al.; Su et al.; Wang et al.; Ciulla et al.; Jeon et al.; Ambrosio et al.; Wang et al.; Bi et al.; Shao et al.; Han et al.; Turco et al.). As evidence of the evolving scenario, it is particularly interesting to note, in this Research Topic, the lack of “classic” studies based on the evaluation of the diagnostic performance of single methods or on the comparison between different imaging methods.

Some studies evaluated the accuracy of imaging in the non-invasive prediction of malignancy in suspicious lesions of the female reproductive organs. In particular, it has been observed that: i) diffusion-weighted MRI (Scepanovic et al.) and multi-parametric (mp) MRI-based radiomic image analysis (Bi et al.) are able to effectively differentiate benign endometrial masses from malignant ones; ii) the US-based ADNEX model can differentiate benign adnexal masses from malignant or borderline ones (Hu et al.); iii) mpMRI can distinguish uterine sarcomas from leiomyomas and atypical leiomyomas (Wang et al.); IV) 3T MRI can predict histological type and grading in cervical carcinomas (Shao et al.). Conversely, mpMRI was unable to predict grading in endometrial tumors (Scepanovic et al.).

In terms of imaging-based prediction of tumor response, one study showed that superb microvascular imaging (an US image processing method) predicts response to chemoradiation in patients with locally advanced cervical cancer (Zhu et al.), while a literature review achieved similar conclusions regarding MRI in the same setting (Matani et al.). Other studies reported the possibility of predicting outcome by imaging in gynecological malignancies. In particular, it has been observed that radiomics analysis of CT and MRI images can predict recurrences in locally advanced cervical cancer (Wang et al.) and that similar results can be achieved, in the same patients, through MRI-based tumor monitoring during treatment (Cordoba et al.), as confirmed by a literature review on the role of MRI in cervical cancers (Matani et al.). Also, the increase in abdominal fat in sarcopenic patients (assessed by CT) during treatment is correlated with a greater risk of recurrences (Han et al.). Conversely, tumor size estimation by US did not correlate with prognosis in endometrial carcinomas (Ambrosio et al.).

Other studies reported on imaging assessments of tumor biology. In particular, it was demonstrated that DCE-MRI is able to effectively evaluate the degree of proliferation (Ki-67) in cervical tumors (Wang et al.), while a literature review analyzed the role of imaging in evaluating the degree of hypoxia in endometrial tumors (deSouza et al.). Interestingly, two papers reported on the role of imaging in predicting or analyzing anticancer drug toxicity. In particular, a systematic review found variable association of body composition by imaging and chemo-induced toxicity in ovarian tumors (Rizzo et al.) while, in a case report, the authors observed that MRI can help distinguish, between two drugs, which one is responsible for the neurological toxicity (Matsuura et al.).

Instead, other studies evaluated the results of integrating different imaging methods. One paper reported a higher sensitivity and specificity, in discriminating adnexal masses, in case of combination of ADNEX model (US) and MRI (Hu et al.), while another study showed better results in the differential diagnosis between uterine sarcomas and benign lesions with the combination of conventional MRI and diffusion-weighted MRI (Wang et al.). In addition, a paper reported effective risk stratification, in patients with cervical cancer, by radiomics analysis based on CT and MRI (Wang et al.). Finally, one study showed that, compared to a single method, the combined CT-based assessment of both waist skeletal muscle volume using an AI-based tool and waist fat gained during treatment improves the prediction of outcomes (Han et al.).

Other studies reported on dynamic imaging assessments. A paper reported reliable prediction of tumor response with US-based superb microvascular imaging monitoring in patients with locally advanced cervical cancer treated with chemoradiation (Zhu et al.). Furthermore, in the same setting, a study showed that tumor monitoring by MRI allows an effective prediction of disease-free survival and overall survival (Cordoba et al.), as confirmed by a review of the literature (Matani et al.). Finally, another literature review on advanced cervical cancer discussed the role of different imaging methods (spectroscopy, PET-MRI, radiomics) in tumor monitoring during radiotherapy and in assessing clinical response (Ciulla et al.).

As further evidence of the growing importance of imaging in the clinical management of patients with gynecological cancers, we must underline the importance of a study evaluating the impact of ^18^FDG-PET-CT in 4167 patients with IB-IVA cervical cancer treated with radiotherapy or chemoradiation. The study, which categorized patients (propensity score-matching) based on whether or not ^18^FDG-PET-CT was performed, showed, regardless of tumor stage, a significant improvement in overall survival in patients undergoing PET (HR: 0.88; _95%_CI: 0.80-0.97; p=0.01) (Su et al.).

We sincerely hope that the papers contained in this Research Topic (Scepanovic et al.; Hu et al.; Wang et al.; Matsuura et al.; Zhu et al.; Rizzo et al.; Cordoba et al.; Matani et al.; deSouza et al.; Su et al.; Wang et al.; Ciulla et al.; Jeon et al.; Ambrosio et al.; Wang et al.; Bi et al.; Shao et al.; Han et al.; Turco et al.) represent a useful update for researchers and health professionals involved in gynecological cancers. Furthermore, we hope that these papers will be a starting point and a stimulus for the design and conduct of further clinical studies in this field. Finally, the growing role of imaging not only in the diagnosis and staging of gynecological cancers, but also in the prediction of the prognosis or even in its refinement, suggest the need for an ever greater integration of imaging in the multidisciplinary management of these neoplasms.

## Author contributions

Both authors have made a substantial, direct and intellectual contribution to the work, and approved it for publication.

